# Giant right atrium, what does it hide? Case presentation

**DOI:** 10.1186/s12872-021-02181-5

**Published:** 2021-08-21

**Authors:** María Martín Talavera, Israel Valverde Pérez, Begoña Manso García

**Affiliations:** 1 Pediatric Cardiology Unit, Hospital Infantil Virgen del Rocío, C/ Torcuato Luca de Tena, 32, 41013 Seville, Spain; 2 Cardiovascular Pathology Unit, Institute of Biomedicine of Seville, Seville, Spain

**Keywords:** Giant right atrium, Right atrium stiffness, Surgical treatment, Case report

## Abstract

**Background:**

Malformation of the right atrium is a rare cardiac abnormality and is usually reported as isolated malformation in the literature.

**Case presentation:**

Prenatal giant atrial dilatation in an asymptomatic infant was treated surgically at 18 months of age, due to potential risk of thrombosis and arrhythmias. Post-surgical echocardiographic images illustrate residual atrial elevated pressure.

**Conclusions:**

Sometimes, as seems in our case, right atrial dilatation hides an associated restrictive right ventricle.

## Background

Malformation of the right atrium is a rare cardiac abnormality and is usually reported as isolated malformation in the literature.

## Case presentation

We present a patient with prenatal diagnosis of right atrial dilatation. Throughout prenatal follow-up, several controls were made observing a normal umbilical vein pattern, normal anterograde flow pattern and normal tricuspid valve without insufficiency until 29-gestation week, when a mild tricuspid insufficiency was observed, which maintains stable during the hole pregnancy. The foramen oval was permeable with right-to-left shunt.

**Fig. 1:**
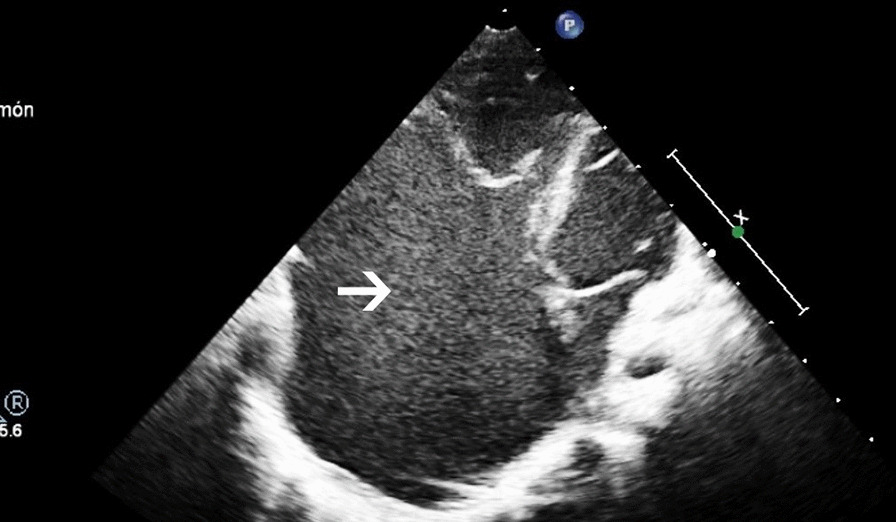
Neonatal echocardiography, 4-chamber plane. The arrow points the right atrial dilatation

Post-delivery, patient was asymptomatic and the diagnosis was confirmed by transthoracic echocardiography (Fig. 1). There were no signs of vascular nor airway compression.

**Fig. 2:**
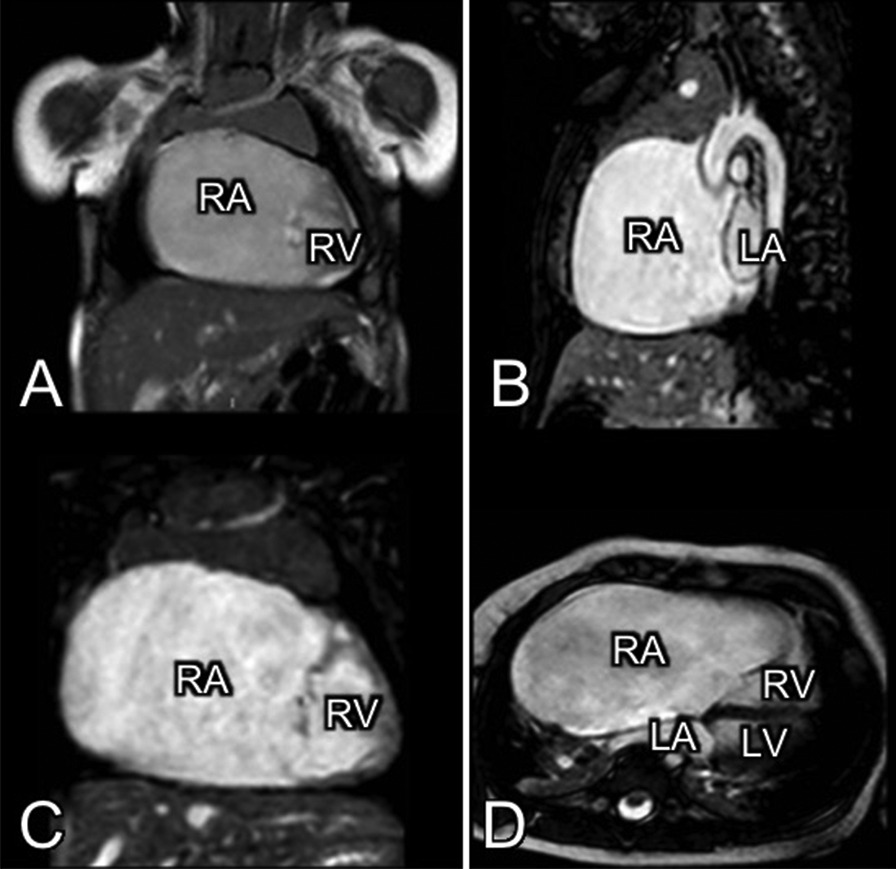
MRI at 6 months. Cardiomegaly with a normal size of the right ventricle. **A**, **C** show a coronal plane, **B** shows a sagittal plane and **D** shows an axial plane. *RA: Right atrium; RV: Right ventricle; LA: Left atrium: LV: Left ventricle*

During follow up, there was a progressive enlargement of the right atrium and the tricuspid valve regurgitation progressed from mild at birth to moderate-severe. Cardiovascular Magnetic Resonance Imaging (MRI) was performed at six months (Fig. 2) showing cardiomegaly at the expense of the right atrium with normal right and left ventricular volumes and function.

Due to the risk of thrombosis and arrhythmias [1], surgical treatment was decided at the age of 18 months. Intraoperative findings confirmed the diagnosis of giant right atrial aneurysm and absence of right atrioventricular sulcus. The right coronary artery was identified in the origin but it could not be identified in the posterior right atrioventricular sulcus and only small collaterals of epicardial vessels were seen over the right ventricle. Extensive right atrium wall resection and De Vega tricuspid valve annuloplasty were performed. At surgery, biopsies of the right atrium wall were taken with the result of unspecific fibrosis. Biopsies of the right ventricle were not taken.

In the course of the immediate postoperative period, a restrictive right ventricle was observed: The interventricular septum bulged from right to left during diastolic phase, the interatrial septum bulged from right to left and the shunt through the foramen oval was right to left predominantly.

**Fig. 3:**
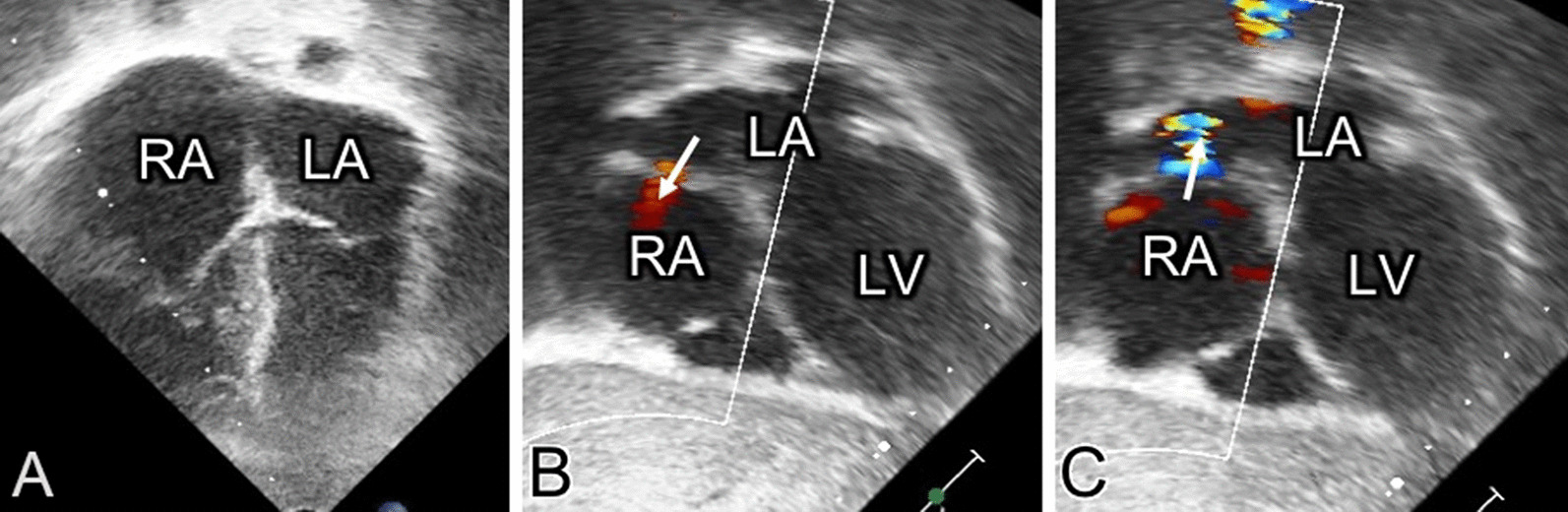
Postsurgical echocardiography. **A** shows an apical-4-chamber plane with convex interatrial septum. **B**, **C** show a subcostal 4-chamber plane. The arrow points the bidirectional shunt in foramen oval. *RA: Right atrium; LA: Left atrium: LV: Left ventricle*

Two years later, the patient remained asymptomatic. Echocardiography study demonstrated a residual right ventricle restriction and indirect signs of elevated right atrial pressures. The right atrium had a globular shape (Fig. 3) with an interatrial septum bulging from right to left and bidirectional shunt across the foramen oval. Evaluation of the inferior vein cava demonstrated reversal ‘a’ wave with normal diameter. The residual tricuspid valve regurgitation was only mild and doppler evaluation excluded the presence of pulmonary artery hypertension.

## Discussion

Giant right atrial aneurysm is an extremely rare condition of unclear etiology [2, 3]. In the absence of predisposing conditions such as congenital heart disease, pulmonary arterial hypertension or tricuspid valve disease, aneurysms of the right atrium are considered to be of congenital origin [4]. The etiology of a congenital aneurysm is unknown. Most patients are asymptomatic and diagnosed after an incidental finding of cardiomegaly on chest radiograph [1]. The most common symptoms are palpitations due to atrial tachycardia, including atrial fibrillation and flutter, dyspnea and syncope [1]. Moreover, thrombo-embolic complications have been reported [4].

The diagnosis of giant right aneurysm is usually established with transthoracic echocardiography. Computer tomography or magnetic resonance imaging may be helpful for a definitive diagnosis [4]. Fetal echocardiography often provides adequate imaging for prenatal diagnosis [1].

The optimal medical and surgical management is still under debate. The majority of patients are asymptomatic, which makes decisions regard to prophylactic surgery and preventative medications difficult [1]. Harder et al. [1] recommend elective surgical intervention for patients with massive right atrial dilatation, severe tricuspid regurgitation due to annular compression, and right ventricular compression. Elective surgery optimally occurs after the neonatal period to avoid risks associated with cardiopulmonary bypass [1].

We have not found enough evidence about the indication of prophylactic treatment regarding to atrial arrhythmias or thrombo-embolisms in asymptomatic patients.

Sometimes, as seems in our case, right atrial dilatation hides an associated primary right ventricle pathology, and the restrictive main right ventricle in fetal life could have caused atrial dilatation. The atrial walls, that can be pathological, have suffered an elevated tension (stress) that maybe has caused its aneurysmal dilatation. The original giant volume of the right atrium cushions the excess of pressure; but the right atrial plasty surgery, reduced the volume of the right atrium to the point of making the persistent high right ventricle end-diastolic pressure, again visible by echocardiography (atrial septum bulging towards left). We resolved a space conflict in the thoracic cage (the right atrial aneurysm) with potential thrombotic and arrhythmic risk, but unmasked a right ventricular restriction, which perpetuates arrhythmic risk and possibly systemic venous ectasia in the future.

The cause of the absence of right atrioventricular sulcus and the absence of the right coronary artery route in posterior atrioventricular sulcus remain unknown for us, as well as the possibility that these findings explain the right atrium dilatation or are just a casual association. To the best of our knowledge, in other giant right atrium cases published so far, these findings were not found.

Nowadays, the patient remains asymptomatic and the right atrium size has been stable since the surgery. Due to the age of the patient, this maintains us on a skeptical position in regards to plan invasive tests, although future MRI and coronary perfusion study are planned to be done when the patient is older.

## Conclusion

Sometimes, as seems in our case, right atrial dilatation hides a problem outside of itself. In spite of surgical right atrium plasty in order to avoid risk factors (arrhythmia, thrombosis), problems derived from underlying right ventricle restriction could be unmasked in follow-up.

## Data Availability

Data and materials analysed during this study are included in the main manuscript and additional supporting files.

## Abbreviation

MRI: Magnetic resonance imaging
